# High in Utero Exposure to Perfluoroalkyl Substances from Drinking Water and Birth Weight: A Cohort Study among Infants in Ronneby, Sweden

**DOI:** 10.3390/ijerph19042385

**Published:** 2022-02-18

**Authors:** Karin Engström, Anna Axmon, Christel Nielsen, Anna Rignell-Hydbom

**Affiliations:** 1 EPI@LUND (Epidemiology, Population Studies, and Infrastructures at Lund University), Division of Occupational and Environmental Medicine, Lund University, 223 62 Lund, Sweden; anna.axmon@med.lu.se (A.A.); anna.rignell-hydbom@med.lu.se (A.R.-H.); 2 Epidemiology, Division of Occupational and Environmental Medicine, Lund University, 223 63 Lund, Sweden; christel.nielsen@med.lu.se

**Keywords:** PFOS, PFHxS, environmental exposure, drinking water contamination, AFFF foam, in utero exposure

## Abstract

In 2013, the drinking water for one-third of the households in Ronneby, Sweden, was found to be contaminated by perfluorinated alkyl substances (PFAS, >10,000 ng/L) from Aqueous Film Forming Foam (AFFF). In utero PFAS exposure can influence birth weight, but little is known about the effects at very high levels. This study aimed to examine the association between in utero PFAS exposure and birth weight. Infants with mothers from Ronneby exposed to contaminated water at home (high exposure) and infants with mothers from Ronneby not exposed to contaminated water at home (low exposure) were compared to infants with mothers from Blekinge county excluding Ronneby (referents). All infants born in Blekinge county 1995–2013 were included (*n* = 30,360). Differences in birth weight were only seen among infants born after 2005. For boys, Ronneby high exposure had a lower mean birth weight than referents (−54 g, 95% CI −97; −11). For girls, Ronneby high exposure had a higher mean birth weight than referents (47 g, 95% CI 4; 90). There were no differences in birth weight between referents and Ronneby low exposure. In conclusion, high exposure to PFAS may influence birth weight in a sex-specific way, although the effect estimates were relatively small.

## 1. Introduction

Perfluorinated alkyl substances (PFAS) are a group of many different synthetic substances produced and widely used for approximately 50 years. PFAS are endocrine-disrupting compounds [1], i.e., they interfere with hormonal biosynthesis and cause developmental disruptions [2]. Associations between PFAS and adverse health effects have been suggested for, e.g., metabolism, thyroid function, neurodevelopment, cancers, cardiovascular diseases, reproductive functions, and immunity [3]. The European Food Safety Authority (EFSA) stresses an increase in serum total cholesterol and a decrease in antibody response at vaccination in children as the critical effects for perfluorooctane sulfonic acid (PFOS), and an increase in serum total cholesterol for perfluorooctanoic acid (PFOA) as the critical effect [4,5].

PFAS are highly persistent compounds found in, e.g., non-stick cookware, impregnated paper and textiles, detergents, and firefighting foams, and can enter the environment through production or waste streams. All humans are exposed to PFAS to some extent through diet, indoor air, and dust [6,7]. In addition, there is exposure from contaminated drinking water in areas with PFAS point-source contamination [8]. The latter was noted as early as the 1990’s around PFAS production facilities and military/civilian firefighting training facilities where large quantities of Aqueous Film Forming Foam (AFFF) were used [9,10]. Exposure can occur in utero as maternal PFAS passes the placental barrier and reaches fetal circulation [11,12]. The average half-lives for the different PFAS are around 2.5–6 years, with marked interindividual variation [13]. Thus, a fetus can be exposed to high levels of PFAS in utero after exposure has ended for the mother. 

The fetal stage is a vulnerable period of development. Birth weight is one of the main indicators of fetal growth and healthy birth outcomes [14]. Although some previous studies suggest that in utero exposure to PFAS is associated with lower birth weight, results have been inconsistent. Whereas some have found associations between PFOA and reduced birth weight [11,15,16,17,18,19,20], others reported no associations [21,22,23,24,25,26,27,28]. Similarly, several studies have found a significant association between PFOS and reduced birth weight [15,17,25,26,29,30,31], whereas others reported no associations [11,18,19,20,21,22,23,24,27,28,32]. The EFSA PFOS and PFOA Opinion [4,5] stated that “human epidemiological studies provide some evidence for a causal association between prenatal exposures to PFOS and PFOA and birth weight”. 

Associations between perfluorohexane sulfonic acid (PFHxS) exposure and birth weight are far less studied than those for PFOS and PFOA and have shown mixed results. Kwon et al. found umbilical cord PFHxS concentration to be negatively associated with birth weight [15], and Maisonet et al. found that serum maternal PFHxS was associated with lower weight in female infants [17]. Other studies did not find any associations between PFHxS and birth weight [16,20,23,24,27,33]. EFSA concluded that maternal serum PFAS levels in studies reporting results on other PFAS than PFOA or PFOS were generally much lower and that there was no evidence for an adverse association for other PFAS than PFOS and PFOA and birth weight [4,5].

So far, most birth weight studies have used exposure assessment relying on measured PFAS in body fluids, mainly maternal serum PFAS concentrations. Studies using maternal serum PFAS concentrations might be confounded by pregnancy-related maternal factors, such as increased glomerular filtration rate [34] and hemodilution [35]. This confounding can be overcome by using a valid proxy measure of exposure. Estimating exposure from register data would be less prone to biases due to physiological changes in pregnancy than studies relying on serum concentrations. Register studies also benefit from being able to include a high number of participants. However, no register studies have evaluated the association between estimated PFOS or PFHxS exposure and birth weight, although previous register studies have assessed the association between estimated PFOA and birth weight [36,37,38]. 

In Ronneby municipality, Sweden, it was discovered that a waterwork supplying a third of the inhabitants was contaminated by per- and polyfluoroalkyl substances originating from firefighting foams used at a nearby military airport where large quantities of AFFF were used [39]. A biomonitoring study showed that exposure to PFOS and PFHxS in Ronneby was high among individuals in areas with contaminated water, while levels of PFOA were lower compared to many other PFAS hotspots [39]. Individuals who resided in Ronneby areas with uncontaminated water also had elevated levels of PFOS and PFHxS [39]. Exposure to PFAS from AFFF is a highly pertinent public health issue, as ongoing environmental monitoring continues to discover new contaminated areas worldwide. AFFF has been used at functioning or closed airports and firefighting training facilities, where there has been widespread leaching of PFAS from the soil to nearby water sources. In Sweden, PFAS contamination has been detected around almost all open or closed airports nationally [40] and in several municipalities with nearby airports (e.g., Visby, Arvidsjaur, and Lulnäset [41,42]). There are still few published studies on general population exposure after AFFF contamination and few reports of health effects [43,44,45]. According to our knowledge, Ronneby has one of the highest known AFFF exposures among the PFAS hotspots studied. 

The Ronneby situation is unique – a "natural experiment" in an area with a homogenous health system, giving the possibility to link estimated exposure from residential addresses, ranging from background to very high exposure, to health register information on birth weight without participation bias. No studies evaluating associations with birth weight have yet been conducted in study groups with similarly high exposure to PFOS and PFHxS. Indeed, for PFHxS, the knowledge about its potential associations with birth weight is still limited.

The current study aims to investigate the association between PFAS exposure and birth weight by comparing groups with significant contrasts in exposure: one group of infants from Ronneby areas with highly contaminated water at home, one group of infants from Ronneby areas without contaminated water at home, and one reference group with background exposure. 

## 2. Materials and Methods

### 2.1. Setting

The waterwork in Ronneby municipality in Blekinge county, which provided drinking water to about 10,000 of the 28,000 inhabitants, was immediately closed when the PFAS contamination was discovered in 2013. The exact starting date of the contamination is still unknown but has been estimated to be in the mid-1980s. More detailed information about the PFAS exposure in Ronneby can be found in Xu et al. [39]. Blekinge is Sweden’s second-smallest province by area, situated in the south of the country. It borders the Baltic Sea, and its population is 158,453 (as of 2016). 

### 2.2. Study Population

All infants born in Blekinge County between 1995 and 2013 were included in this study. (*n* = 46,221 infants). There were no exclusion criteria. However, we included only complete cases in the analyses, i.e., those which had data on exposure, outcome, and all variables considered confounders and effect modifiers (*n* = 30,360). The study was approved by the Regional Ethical Review Board in Lund, Sweden (approval number: 2014/4, 2017–437).

### 2.3. Register Data

Information on birth weight, maternal smoking, height and weight in early pregnancy, gestational age, and maternal age were collected from the Swedish Medical Birth Register, maintained by the Swedish National Board of Health and Welfare. Data regarding maternal education from 1990 onwards were collected from the Education Register at Statistics Sweden. 

### 2.4. Exposure Assessment

For the participants from Ronneby, in utero exposure was determined by maternal exposure during a five-year period before the birth of the infant. Maternal exposure was defined based on residential address on 31 December 31 each year. By linking residential address on 31 December 31 each year 1990–2013 to data on yearly water supply obtained from the municipal water company, we obtained yearly data on whether a residential address was provided with uncontaminated or contaminated municipal water. Extensive measurements in private wells in the municipality did not reveal any high PFAS levels [39], and residents with private wells were thus considered to have uncontaminated water. Additionally, all other municipalities in Blekinge county had uncontaminated water. 

Infants were categorized as either Ronneby high exposure: exposed to contaminated water at home (mother had been exposed to contaminated water at least one year of the five years before the birth of the infant), Ronneby low exposure: not exposed to contaminated water at home (mother had been living in Ronneby but had not been exposed to contaminated water) or reference group: participants from Blekinge county excluding Ronneby, i.e., with a background exposure. 

### 2.5. Statistics

Birth weight in the three groups was compared using Analysis of Variance (ANOVA). All analyses were stratified by infant sex. Since PFAS exposure was suspected to be higher the decade before the exposure stopped, period (before or after 2005) was assessed as a potential effect modifier by introducing an interaction term between period and exposure group. 

Adjusted models included parity (1, 2, 3+), maternal education (pre-secondary, secondary up to 2 years, secondary 3 years, college/university), maternal smoking in early pregnancy (non-smoker, 1–9 cigarettes per day, or 10+ cigarettes per day), maternal age (continuous), gestational age (continuous) and BMI in early pregnancy (continuous). These variables were chosen based on a priori knowledge of variables potentially confounding the association between PFAS and birth weight.

The analyses were run as complete cases, i.e., only including births with complete data on exposure, outcome, effect modifiers, and confounding variables. We performed sensitivity analyses based on imputed datasets to check the impact of exclusion due to missing data. Imputation was performed using the multiple imputation command in SPSS, performing 20 imputations including exposure group, outcome (birth weight) and all confounding variables in the model. The fully conditional specification method with ten iterations was used. Scale variables were imputed using linear regression, and categorical variables using logistic regression. 

All analyses were performed in IBM Statistics SPSS 27.

## 3. Results

**Figure 1:**
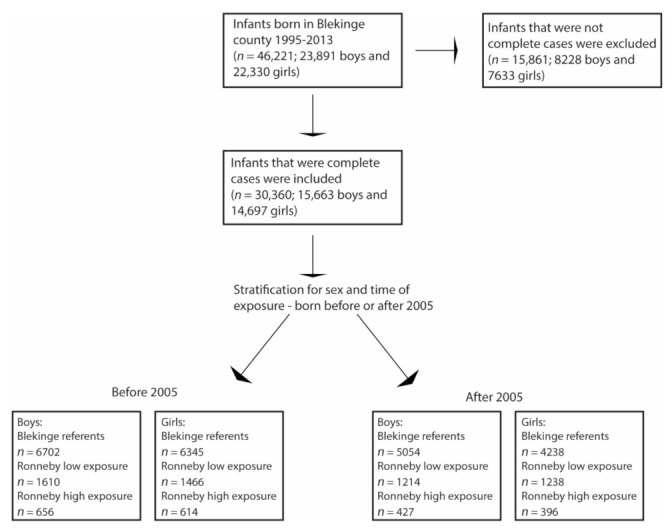
Flowchart of the participant selection and grouping.

Background characteristics are presented in Table 1 and Table 2. A total of 46,221 infants were born in Blekinge county 1995–2013 (23,891 boys and 22,330 girls). Of these, 30,360 had complete information and were included in the analyses. The interaction term between period (born before or after 2005) and exposure group was statistically significant for boys when comparing Ronneby high exposure and referents (*p* = 0.002), but not for girls (*p* = 0.068) and not when comparing Ronneby low exposure and referents (*p* = 0.50 for boys and *p* = 0.18 for girls). As one interaction was statistically significant, we chose to stratify all analyses for period (17,393 were born before 2005, and 12,967 were born after 2005). A flowchart of the participant selection and grouping is shown in Figure 1. Compared to the two other groups, the mothers in the Ronneby high exposure group had lower mean age, higher BMI in early pregnancy, a lower level of education, and a higher percentage of the mothers were smokers. 

**Figure 2:**
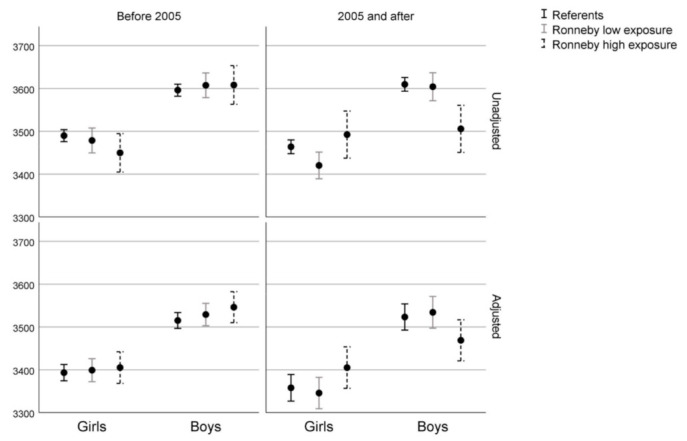
Mean birth weight with 95% confidence intervals stratified for infant sex and period (before or after 2005). Adjusted models include parity, maternal civil status, education level, maternal age, gestational age, smoking habits and BMI in early pregnancy.

**Figure 3:**
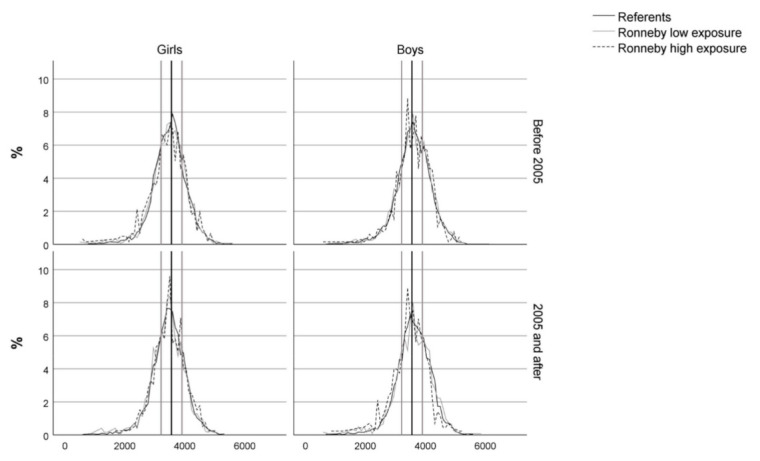
Distribution of birth weight stratified for infant sex and period (before or after 2005) for the different exposure groups. The black lines represent the median, and the surrounding grey lines represent quartiles.

Statistically significant differences in birth weight between referents and the Ronneby groups were only seen among infants born after 2005. Figure 2 depicts the birth weight distribution for boys and girls before and after 2005 and shows that no significant shifts in birth weights could be seen before or after 2005. For boys born after 2005, the Ronneby high exposure group had a lower mean birth weight than referents (−54 g, 95% CI −97; −11) Figure 3. In contrast, for girls born after 2005, the Ronneby high exposure group had a higher mean birth weight than the Blekinge referents (47 g, 95% CI 4; 90). No differences were seen between Ronneby low exposure group and referents. In the sensitivity analyses using imputed data, the number of infants was increased to 46,221. The results in the sensitivity analyses were similar to the analyses of the complete cases dataset (data not shown).

## 4. Discussion

This study shows that high exposure to PFAS may influence birth weight in a sex-specific way. However, the effects were relatively small considering the significant contrasts in exposure between the reference group (background exposure) and the Ronneby high exposure group. We found that boys born to mothers residing in areas with very high PFAS exposure in drinking water had a decreased birth weight compared to boys from the reference group. The association was in the opposite direction for girls, where those with mothers residing in areas with very high PFAS exposure in drinking water had increased birth weights compared to girls from the reference group. 

To the best of our knowledge, this is one of the largest studies evaluating the association between PFAS exposure and birth weight. Additionally, many birth weight studies have been performed at background exposure levels where the results cannot be extrapolated to high exposed groups. Further, the knowledge about the influence of PFHxS exposure on birth weight is still limited, and no other studies have evaluated the influence of PFAS exposure on birth weight in a cohort with a firefighting foam exposure profile with high PFOS and PFHxS. Ongoing environmental monitoring continues to discover new contaminated areas around the world. Our study thus addresses a research question that warrants immediate attention to safeguarding the health of the next generation in AFFF-exposed communities. 

The PFAS-exposed Ronneby population is uniquely exposed, with high and long-term exposure to PFAS. Associations with birth weight were only seen for children born after 2005, which can be an effect of higher exposure levels stemming from higher cumulative maternal exposure and higher levels of PFAS in the contaminated aquifer the decade before exposure was detected [39]. There were no differences in birth weight between the reference group (background exposure) and the Ronneby low exposure group, although previous biomonitoring studies from 2013 (*n* = 2219, both sexes included, ages 0–92 years) have shown that individuals living outside exposed areas in Ronneby had a considerably higher serum PFAS than a reference group with background exposure [39]. The elevated levels for individuals living outside exposed areas in Ronneby are probably partly due to drinking contaminated water at work, at school, or during visits to the areas supplied by the contaminated waterworks. Individuals living outside exposed areas in Ronneby also had higher serum PFAS than many groups considered to be exposed in previous studies evaluating the associations between PFAS exposure and birth weight [4,5], for which the average maternal serum PFOA levels ranged between 0.9 ng/mL [15] and 31.0 ng/mL [26], and serum PFOS ranged between 0.6 ng/mL [15] and 35.3 ng/mL [11].

PFAS levels in drinking water in Ronneby have previously been measured from contaminated waterworks (PFOS 8000 ng/L, PFOA 100 ng/L, PFHxS 1700 ng/L), minimally contaminated waterworks outside the exposed area (PFOS 27 ng/L, PFOA 1 ng/L, PFHxS 4.6 ng/L), and from the main waterworks in Karlshamn, another municipality in Blekinge county (PFOS < 0.2 ng/L, PFOA < 0.3 ng/L, PFHxS < 0.3 ng/L) [39]. The average serum PFAS levels for people that resided in Ronneby, in the area with water supply from the highly contaminated waterworks during the years before the exposure stopped, were 13 ng/mL for PFOA, 239 ng/mL for PFOS, and 210 ng/mL for PFHxS [39]. For Ronneby residents living outside the exposed areas, the average levels were 3.5 ng/mL for PFOA, 40 ng/mL for PFOS, and 30 ng/mL for PFHxS [39] and for a study group in Karlshamn, a neighboring municipality without PFAS in drinking water, the average levels were 1.5 ng/mL for PFOA, 3.9 ng/mL for PFOS, and 0.84 ng/mL for PFHxS [39]. It should, however, be noted that these mean values are for groups comprising both sexes and all ages. Women of fertile age generally have lower PFAS levels due to, e.g., menstruation, pregnancy, and breastfeeding [39]. Thus, the average serum PFAS values for women of fertile ages were about half of the average serum PFAS values for all persons from Ronneby (in both exposed areas and outside exposed areas) included in the study [39].

Previous studies at lower exposure levels have shown that exposure to PFAS is associated with lower birth weight [4,5,15]. However, little is known about the associations between PFAS and birth weight at exposure levels as high as those in Ronneby. If the dose–response between PFAS and birth weight were linear, the large difference in exposure contrasts in the present study would result in larger differences in birth weight than those from studies of a narrower exposure range. Although the exposure range between referents with background exposure and Ronneby high exposure is known to be large, the differences in birth weight between those groups were only around 50 g. Since previous studies have shown that individuals with high exposure in Ronneby have average levels of PFOS of around 200 ng/mL, and women of fertile age had approximately half of these average levels [39], the changes in birth weights seen in our study give us rough estimates of around −0.50 g (boys) and +0.50 g (girls) change per ng/mL increase in serum PFOS, respectively. Previous meta-analyses of epidemiologic studies have suggested that each nanogram per milliliter increase in maternal serum PFOS concentrations was associated with reductions in birth weight of −0.92 g (95% CI −3.4; 1.6) [46] and −3.22 g/ng/mL (95% CI −5.11, −1.33) [47]. There is a complexity in evaluating associations between maternal serum during pregnancy and birth weight: a previous meta-analysis [48] showed that when a term for the timing of blood draw was included in the model, there was essentially no relationship between birth weight and PFOS, and when using serum sampled early in pregnancy, little or no association was found between PFOA and birth weight. However, an association was found when using serum sampled later in pregnancy. The clinical relevance of the small shift in birth weight in the present study is uncertain. Although it is not of importance to the health of an individual in most cases, it may be a marker of moderate toxicity, and it is important to note that even a small numerical difference can be problematic since it implies a shift in the population.

This study showed sex-specific effects of PFAS exposure on birth weight. Since PFAS is an endocrine disruptor, which interferes with hormonal biosynthesis, sex-dimorphic effects are likely to occur from their endocrine-disrupting effects [2]. Fetal growth is primarily mediated by the placenta. The placentas also show altered adaptive responses to stressful environments depending on the sex of the fetus [49]. Few studies have evaluated the association between PFAS exposure and birth weight in a sex-stratified material. Lind et al. [32] found a tendency of maternal PFOS to be associated with reduced birth weight in boys and increased birth weight in girls, although these differences were not statistically significant. In contrast, Kishi et al. [50] and Washino [25] found a negative association between maternal PFOS levels and birth weight for girls but not for boys.

Exposure estimates based on the mother’s residency are subject to error and could lead to exposure misclassification. Reasons for misclassification can be, e.g., that exposure can take place at other places than the residency or that the mothers use bottled water instead of tap water (although in Sweden, the water quality is generally high and complete replacement of tap water with bottled water is rare). It has previously been shown that there are considerable contrasts in serum PFAS between the groups classified as Ronneby low and high exposure [39], and the risk of misclassification should thus be low. There may also be advantages to using exposure estimates based on residency: as individual exposure measurements are more removed, the exposure classification becomes less susceptible to biases from confounding by factors that can often be difficult to control, such as changes in kidney function and behavior [51]. Estimates based on residency are also less susceptible to reverse causation since plasma volume expansion during pregnancy is associated with birth weight and likely also with reduced serum PFAS levels [48].

## 5. Conclusions

In conclusion, this study shows that high exposure to PFAS may influence birth weight in a sex-specific way. However, the effect estimates were relatively small considering the high contrasts in exposures compared to other studies on birth weight. This implies that high exposure to PFAS may have a minor influence on birth weight.

## Figures and Tables

**Table 1:** Descriptive data by exposure group and period, categorical variables.

Variables/Categories	Blekinge Referents		Ronneby Low Exposure		Ronneby High Exposure	
	Before 2005	2005 and after	Before 2005	2005 and after	Before 2005	2005 and after
	*n* (%)	*n* (%)	*n* (%)	*n* (%)	*n* (%)	*n* (%)
Education						
Pre-secondary (any)	1482 (11)	967 (10)	339 (11)	249 (10)	236 (19)	131 (16)
Secondary (up to 2 years)	4368 (33)	935 (10)	977 (32)	221 (9)	520 (41)	124 (15)
Secondary (3 years)	2922 (22)	3026 (31)	692 (22)	739 (30)	284 (22)	308 (37)
College/university (incl graduate)	4275 (33)	4764 (49)	1068 (35)	1243 (51)	230 (18)	260 (32)
Sex						
Male	6702 (51)	5054 (52)	1610 (52)	1214 (50)	656 (52)	427 (52)
Female	6345 (49)	4638 (48)	1466 (48)	1238 (50)	614 (48)	396 (48)
Parity						
1	5323 (41)	4082 (42)	1373 (45)	1171 (48)	544 (43)	367 (45)
2	4937 (38)	3683 (38)	1103 (36)	875 (36)	475 (37)	275 (33)
3+	2787 (21)	1927 (20)	600 (20)	406 (17)	251 (20)	181 (22)
Smoking						
Non-smoker	11,251 (86)	8971 (93)	2610 (85)	2259 (92)	974 (77)	676 (82)
1–9 cigarettes/day	1243 (10)	582 (6)	308 (10)	145 (6)	186 (15)	107 (13)
10+ cigarettes/day	553 (4)	139 (1)	158 (5)	48 (2)	110 (9)	40 (5)

**Table 2:** Descriptive data by exposure group and period, continuous variables.

Variable/Values	Blekinge Referents		Ronneby Low Exposure		Ronneby High Exposure	
	Before 2005 (*n* = 13,047)	2005 and after (*n* = 9692)	Before 2005 (*n* = 3076)	2005 and after (*n* = 3452)	Before 2005 (*n* = 1270)	2005 and after (*n* = 823)
BMI (kg/m^2^)						
Median	23.42	24.22	23.11	24.03	23.48	25.04
25%	21.45	21.89	21.16	21.72	21.48	22.05
75%	26.23	27.55	25.54	27.34	26.50	29.02
Maternal age						
Median	29	30	28	29	27	28
25%	25	26	25	26	24	24
75%	32	33	32	33	30	32
Gestational age, days						
Median	280	279	280	279	279	278
25%	272	272	272	272	271	270
75%	286	286	286	286	285	285

## Data Availability

The data presented in this study are available on request from the corresponding author. The data are not publicly available due to restrictions in privacy.
